# Comparison Between Family Power Structure and the Quality of Parent-Child Interaction Among the Delinquent and Non-Delinquent Adolescents

**DOI:** 10.5812/ijhrba.13188

**Published:** 2014-06-08

**Authors:** Anahita Khodabakhshi Koolaee, Hossein Shaghelani Lor, Ali Akbar Soleimani, Masoumeh Rahmatizadeh

**Affiliations:** 1 Department of Counseling, Science and Research Branch of Arak, Islamic Azad University, Arak, IR Iran; 2 Department of Psychology, University of Science and Culture, Tehran, IR Iran

**Keywords:** Parenting, Parent-Child Relations, Delinquency

## Abstract

**Background:**

Few studies indicate that most behavioral problems are due to family dysfunction and inappropriate family environment. It seems that the family of the delinquent adolescent is unbalanced in the power structure and parenting style.

**Objectives:**

The present study compares the family power structure and parent-child relationship quality in delinquent and non-delinquent young subjects in Tehran.

**Patients and Methods:**

Eighty students of secondary schools aged between 15 and 18 in Tehran were enrolled with cluster sampling method and 80 delinquent adolescents of the Correction and Rehabilitation Centers aged between 15 and 18 were chosen with a convenience sampling method. They responded to an instrument of family power structure (Child–parents relationship inventory). Data was compared between these two groups by utilizing the independent and dependent t-test and Levene’s test.

**Results:**

The findings indicated there is a significant difference between delinquent and non-delinquent adolescents in family power structure and its subscales (P < 0.001) and father-child relationship quality (P < 0.005). Also, there is no statistically significant difference between these two groups in mother-child relationship quality (P < 0.005). Besides, the results revealed that delinquent adolescents were significantly different regarding the quality of parent-child relationship (P < 0.001).

**Conclusions:**

These results emphasize that an inappropriate decision making process pattern in a family has a significant effect on deviant behavior in adolescents. The fathers’ parenting is more strongly linked to their sons’ delinquency. So, family power structure and parent-child relationship can be considered in therapeutic interventions (prevention and treatment) for adolescents’ delinquency.

## 1. Background

The period of adolescence is a period of major physical, cultural, cognitive, and psychological changes. Studies reveal that delinquent behaviors of non-violent and violent types increase in the late adolescence (age 17-18) (1). There is an agreement that deviant behavior in adolescents would lead to an elevated likelihood of adult criminal behavior (2). Teenage delinquents are those whose adaptation to issues and situational challenges arising in a transitional developmental process from childhood to adulthood results in serious issues with parents, psychological problems, and problems such as anxiety and depression and behavioral problems, including drug use or antisocial activities (3). There is substantial evidence to suggest that the family has an essential role in the development of adolescent delinquent behavior (4). Disrupted parental attachment, parental over-control, poor relationships with parents, poor supervision, poor role models in issue resolving, contradictory control by parents, family instability, poverty, and accessibility to financial resources are among the family factors which have been linked to delinquency (4-7). In other words, information regarding the type of associations within the family, such as family flexibility, cohesion and satisfaction offers additional information regarding the youths behavior (8, 9). Different scientific schools of thought regarding family systems theory have already been formed that offer particular methods and terminology for assessing and answering the complicated familial and contextual impacts on childrens’ psychological, emotional and behavioral problems. Tenets from one particular program, Structural Family Therapy (SFT), appear to possess specific application as a framework to enhance our understanding of the systemic antecedents of violence among young ones (10). In accordance with the principles of structure family therapy (SFT), “family structure” identifies recurring relationship patterns within a family group that determines if family unit members connect to each other or the exterior world, in what actions do the members participate and how the functions of each member are linked to the influences of the external systems (11). Therefore, this theory emphasizes the role of power structure within a family group identified as the amount of impact that every member of the family has on family function and decision-making (12). Ultimately, family power is arranged within a multi generation hierarchy where parents and other adults with the principal responsibility for child rearing posses the ultimate power in making family decisions and setting out the principles for children. The transfer of the power and responsibility to children is then coordinated in particular according to age of children in order to achieve the most accomplishments and enhancing the childrens’ self-esteem by ensuring that expectations will not exceed the capabilities (13). From the structural theory viewpoint, a dysfunctional family system exists when issues are more than one of the hierarchical, and boundary or alignment elements of its structure have impaired its resources for coping with and adapting effectively to contextual stressors (13). A dysfunctional hierarchy occurs when parents neglect exercising their authority and responsibilities in the family (10). The causes of parents' failure to use their authority in a family group are numerous; nevertheless, substance abuse, psychological problems, being too young, marital discord, occupational problems, and insufficient parenting skills. Whatever the cause of defective parental authority in the family group is, it may predispose to violence in children particularly when it involves neglect or abuse or violence modeled through the spousal or parental relationships and thus children will incorporate violence in their behaviors (8). Previous studies have suggested that styles of family decision making and family structure both have an important impact on adolescents’ deviance behavior. (3) Satir implies that in a family group wherever parents communicate with their children with an uncertain and obscured attachment style, parents may have a low level of self-worth and use children for their own values. In such cases, adolescents are predisposed to adjustment disorders, including delinquency or psychological problems (3). Studies suggest that the cohesiveness of the family effectively anticipates the frequency of delinquent behavior in non-traditional families (8, 9). As discussed earlier, one of the family factors that impact delinquency is parenting skills. Also, parenting skills has been identified as the best predictors of criminal behavior among other family traits (2, 5, 14, 15). Parenting styles could be referred to as patterns of behavior that principal caregivers use to communicate with their children (16). Baumrind discussed demanding and responsiveness as two independent measures of parenting skills (17). Demanding describes the level that parents display control, power assertion, maturity, and directing. Responsiveness identifies the level that parents would display their warmth, emotional expression, approval and support towards their children. On the basis of the level of parental demanding and responsiveness, four parenting styles have already been identified: authoritative (both demanding and responsive), authoritarian (demanding however not responsive), permissive (responsive however not demanding), and neglectful (neither demanding nor responsive). Each style of parenting is thought to deferentially impact children’s academic outcomes (14, 17). Harsh and irregular parenting is a key cause of conduct disorders (5, 14). Some specific styles of parenting are shown to precipitate delinquency among adolescents which include too strong control, parental disharmony, rejection of the child and insufficient engagement in the child's actions (18). The neglected adolescent is highly likely to become a drug abuser, tough criminal, aggressive, restive, thief, cultist, rapist, etc. The parental monitoring and control of the adolescent’s behavior might be limited due to the financial problems of the parent and family (14, 19, 20). As noted above, the family is the primary role player in child’s development and a reduction in antisocial and delinquent behaviors. Several research studies have been performed on the factors which affect juvenile delinquency, nevertheless still a gap exists regarding the precursor familial factors such as family power structures which affect delinquency. Thus, we tried to investigate this subject further. 

## 2. Objectives

Based on the above background, the current study aims to compare family power structure and parent-child relationship quality between delinquent and non-delinquent adolescents in Tehran.

## 3. Patients and Methods

### 3.1. Participants and Study Design

This study was conducted in 2012 in Tehran; the present study is a causal-comparative study. The sample consisted of two groups: 80 adolescents aged between 15 and 18 residing in the Tehran Juvenile Correction and Rehabilitation Centers and a control group composed of 80 adolescents aged between 15 and 18; the control group was selected with cluster sampling method from secondary schools of Tehran. The delinquent group were selected through the convenience sampling method from the Tehran Juvenile Correction and Rehabilitation Centers. The inclusion criteria were as follow: Age ranged between 15 and 18 years, Any levels of reading and writing ability were considered, Without any severe mental and physical illnesses, Living with both birth parents.

### 3.2. Measurements

#### 3.2.1. Sociodemographic Data Sheet

A sociodemographic data sheet was used to record personal information of the delinquent adolescents including age, education, birth order and the personal information of the mother and father of the adolescent including age, education and job type.

#### 3.2.2. Child–Parents Relationship Inventory

The basic form of this questionnaire was designed by Fine et al. (21), with the aim of evaluating the quality of children-parent relationship. This inventory is a 24 item self-report measure that focuses on the adolescents and their relationships with their parents. The parent-child relationship test includes two forms: the first one evaluates mother-child relationship and the second one evaluates the father - child relationship. The Cronbach alpha reliability for subscale of father is between 0.89 and 0.94 and for subscale of mother is between 0.61 and 0.94. The Cronbach alpha reliabilities for the Persian version of the scale are reported as well: subscale of father = 0.93, subscale of mother = 0.92.

#### 3.2.3. Family Power Structure

This questionnaire was designed by Saidian (22). This inventory is a 63-item self-report measure. The family power structure contains three subscales: couple-related family power, family power structure, and the method of enforcement of couple power. The maximum and minimum scores in subscale of the family power structure are 230 and 46, so that higher scores reflect greater power structure in the family. Participants respond to items on a five-point Likert-type scale, ranging from one (not at all true for me) to five (very true for me). The original study reported internal consistency reliabilities (Cronbach alpha) as follow: couple-related family power = 0. 83, family power structure = 0.85, the method of enforcement of couple power in the family = 0.73.

### 3.3. Procedure

All mothers were asked to complete the Family Power Structure questionnaire. Students were asked to complete the Child–Parents Relationship Inventory and Sociodemographic data sheet. Then collected data was analyzed using SPSS-13 software. Data was compared between these two groups utilizing independent t-test, dependent t-test and Levene’s test.

## 4. Results

In Tables 1 and 2, the results of socio-demographic characteristics of all participations are shown. As shown in Table 1, the highest category of age in delinquent was at 16 (42.5%) and in non-delinquent group was at 15 years (35%). Also the most delinquent adolescents were the second child in the family (26.25%) and the most non- delinquent adolescents were the first child in the family (37.5%). The highest category of education level in the delinquent group was secondary school (52.5%) and in non -delinquent group was the nineth grade (31.25%).

**Table 1.** Socio-Demographic Characteristic of Delinquent and Non-Delinquent Adolescents (Percentage) ^a^

	Absolute Frequency (n_i_)	Cf	Age Group, y	Absolute Frequency (n_i_)	Cf	Birth Order	Absolute Frequency (n_i_)	Cf
**Non-delinquent Juvenile**								
9th grade	25	31.25	15	28	35	1	30	37.5
10th grade	20	25	16	20	25	2	23	2875
11th grade	20	25	17	20	25	3	18	22.5
12th grade	15	18.75	18	12	15	4-6	9	11.25
**Delinquent Juvenile**								
Elementary school	27	33.75	15	22	27.5	1	16	20
Secondary school	42	52.5	16	34	42.5	2	21	26.25
High school	11	13.75	17	13	16.25	3	24	30
High school	11	13.75	18	11	13.75	4-6	19	23.75

^a^ Abbreviations: df, degree of freedom; Cf, cumulative frequency.

As shown in Table 2, the highest fathers’ education level of delinquent adolescents was the secondary school (46.25%) and in non-delinquent group was the high school (45%). Also, this frequency in mothers of delinquent adolescents was the high school (41.25%), and for the mothers of non-delinquent adolescents was high school (43.75%). The most frequent job of fathers of delinquent adolescents was self-employment (52.5%) and for fathers of non-delinquent group was employee (57.5%). Also the most frequent job of mothers of delinquent adolescents was housewife (88.75%) and it was the same for the non-delinquent group (78.75%). Table 3 provides the means, standard deviations and results of Levene’s test and t test of all the variables used in this study.

**Table 2.** Socio-Demographic Characteristic of the Parents (Percentage)

	Non-Delinquent Adolescent		Delinquent Adolescent	
	n_i_	Cf	n_i_	Cf
**Level of education of the father**				
Illiterate	0	0	4	5
Elementary school	13	16.25	14	17.5
Secondary school	17	21.25	37	46.25
High school	36	45	20	25
University	14	17.5	5	6.25
**Level of education of the mother**				
Illiterate	0	0	6	7.5
Elementary school	11	13.75	14	17.5
Secondary school	15	18.75	18	22.5
High school	35	43.75	33	41.25
University	19	23.75	9	11.25
**Father job status**				
Employee	46	57.5	38	47.5
Self-employment	34	52.5	42	52.5
**Mother Job Status**				
Employed	17	11.25	9	11.25
Non employed	63	78.75	71	88.75

**Table 3.** Mean, SD and t Value of Parent-Child Relationship Quality and Family Power Structure for Delinquent Adolescent (Group 1, n = 80) and Non-Delinquent Adolescent (Group 2, n = 80) Groups ^a, b^

	Results	Levene’s Test		t-test		
		P Value	F	df	P Value	t Value
**Quality of child-father relationship**		0.784	0.076	154.41	0.003 ^c^	2.866
1	92.57 ± 21.72					
2	103.31 ± 25.53					
**Quality of child-mother relationship**		0.225	1.481	158	0.581 ^c^	1.0801
1	104.68 ± 17.87					
2	107.77 ± 18.27					
**Family power structure**		0.001	10.442	145.925	0.000 ^d^	7.927
1	53.78 ± 109.83					
2	61.66 ± 7.68					
**Family power couple related family power**		0.008	7.187	148.832	0.000 ^d^	3.632
1	88.24 ± 15.54					
2	96.22 ± 12.06					
**Method of couple power enforcement within family**		0.007	7.536	5.256	0.000 ^d^	5.256
1	20.05 ± 6.85					
2	27.61 ± 5.09					
**Family power structure's total score**		0.001	12.792	141.031	0.001 ^d^	6.223
1	162.60 ± 27.29					
2	185.74 ± 19.01					

^a^ Abbreviations: SD, standard deviation; df, degree of freedom; t, student's t-test.

^b^ Data are presented as Mean ± SD.

^c^ P < 0.005.

^d^ P < 0.001.

The results of Levene’s test show that variance of family power structure variable and its subscale in two groups is unequal so that t-test is used. These results are displayed in Table 3 which shows that there is a significant difference between family power structure and its subscales in two groups (family power structure's total: t = 6.223,P = 0.001, df = 141. 031; couple-related family power: t = 3. 632, P = 0. 000, df = 148. 832; family power structure: t = 5. 256, P = 0. 000, df = 141. 352; the method of couple power enforcement within the family: t = 7. 927, P = 0. 000, df = 145. 925). According to Levene’s test, the variables’ variance in child-father relationship quality in two groups is unequal, so, there is a significant difference between child-father relationship quality in two groups (t = 2. 866, df = 154. 41, P = 0. 003). Also the result of Levene’s test shows that the variances’ of quality of child-mother interaction is equal and the variable variance of child-mother relationship quality variance in two groups is equal (t = 1. 0801, P = 0. 581, df = 158). The results shown in Table 4 implies that there is a significant difference between the child-father and child-mother relationship quality in delinquent group (t = -5.715, P = 0.000, df = 79) but there is not a significant difference between child-father and child-mother relationship quality in non-delinquent group (t = -1.919, P = 0.059, df = 79).

**Table 4.** Results of Dependent t-test Value of Parent-Child Relationship Quality in Delinquent and Non-Delinquent Groups

	Variable	df	P Value ^a^	t
**Delinquent**	quality of child-mother relationship	79	0.000	- 5.715
	quality of child-father relationship			
**Non-delinquent**	quality of child-mother relationship	79	0.059	- 1.919
	quality of child-father relationship			

^a^ P < 0.001.

## 5. Discussion

The juvenile delinquency is a really complicated problem and there are nearly as much expected reasons for delinquency. This study was performed to determine the differences in family power structure and parent-child relationship quality in delinquent and non-delinquent adolescents aged between 15 and 18. As shown in the Table 3, the average of family power structure score in delinquent (162.60) is less than non-delinquent group (185.74). So, the findings of the present study is similar to previous findings which indicate that lower level of family power structure is associated with behavioral delinquency in adolescents. For example, Chedid et al. (23) showed weak hierarchic relations with big ‘‘intergenerational coalitions’’ within the families. The non-equilibrated family structure (extreme levels of cohesion and power) could be more significantly correlated with marijuana consumption behavior can considerably correlate with marijuana use (23). These findings are in line with previous studies revealing that family disruption contributes to delinquency (2, 6). The subject of disrupted family structure as a one of the cores delinquency theory is widely agreed upon (24). Review articles suggest that the risk of delinquency is twice more for children from disrupted family structures in comparison to those from healthier families, and this effect has been consistently shown across times and locations (6, 25, 26). Previous data indicates that adolescents that live in a single parent home are more likely to participate in deviant behavior. Moreover, demonstrating a different decision making process in a single parent household significantly influences the deviant behavior in adolescents (8, 9, 27, 28). Infrequent communication between two parents and the strength of child and parent bond were found to be associated with higher rates of deviance behavior (9, 25). It can also be argued that family structure itself might have significant effects on the degree of power individual family members and extent of control on adolescents (28). Therefore the results of the current study suggest that disrupted parenting practices are causally related to childrens’ antisocial behavior. Additional research also suggests that family structure, although important is not as important as parenting methods (2). Likewise, the results of this survey indicate that there is significant difference between father-child relationship quality in delinquent and non-delinquent adolescents. Contrary to these results, some previous studies have shown that poor paternal support is much more detrimental than poor maternal support, especially for sons (5, 15, 29). For instance; Chedid et al. revealed that the lack or even a poor connection with the father could predispose to marijuana use (23). On the other hand, Moitra et al. had found that having adequate mother-adolescent interaction is more essential than father-adolescent interaction (4). It could be argued that whenever one parent was neglectful, the level of delinquency of his or her child was dependent on the parenting style of the other parent. As an example, having two neglectful parents was linked to higher degrees of delinquency, whilst having one neglectful parent was not. That implies that fathers’ parenting might compensate for the neglectful parenting behavior of the mothers or vice versa (5). On the other hand, in accordance with family system theory, the disease symptoms play an important role in maintaining stability in the family (13). Bowen identifies that the common pattern in couples with conflict is a pattern of closeness followed by a conflict which produces distance, and often is followed by the couple resuming to be the extremely close to each other. In a fused relationship, partners consider understanding the emotional state of another one as their responsibility but regard the disagreement as a personal affront. The child with the least emotional separation from his/her parents is considered the most vulnerable to delinquency. Bowen considers delinquency as the childrens’ anxious reaction to the stress already present in the parental relationship. A detouring triangle is hence formed as attention and protectiveness are transferred to the child. Through this pattern of reciprocal anxiety, a child becomes more demanding or even more impaired. An illustration could be given when a disease in a child distracts one parent from the pursuit of closeness in the marriage. As tension in the marriage is treated, both spouses become committed to treating their child's situation, which might consequently become serious or psychosomatic (12, 13). Based on these results and previous studies, we suggest that parenting styles and family power structure probably represent an important target of interventions among delinquent adolescents. It could be beneficial for practitioners to attempt involving both fathers and mothers in the therapy programs. One of the limitations of this study is confinement of the research items to the ones related to the mothers. 
